# Impact of smoking behaviors on asthma incidence and allcause mortality in middle-aged and older adults: A longitudinal study from China

**DOI:** 10.18332/tid/207912

**Published:** 2025-09-09

**Authors:** Tingting Fu, Shilong Zhao, Chunling Hu, Jing Gao, Lihua Xing

**Affiliations:** 1 Department of Respiratory and Critical Care Medicine, The First Affiliated Hospital of Zhengzhou University, Zhengzhou, China

**Keywords:** smoking behaviors, adolescent smoking initiation, asthma incidence, middle-aged and older adults, all-cause mortality

## Abstract

**INTRODUCTION:**

The impact of smoking behaviors on asthma incidence and all-cause mortality among middle-aged and older adults remains understudied. In particular, whether the potential effect of adolescent smoking initiation on late-onset asthma is independent of cumulative tobacco exposure is unclear.

**METHODS:**

Cox proportional hazards models assessed longitudinal impact of smoking behaviors on asthma incidence and mortality risks using 2011–2018 China Health and Retirement Longitudinal Study (CHARLS) data. Cross-sectional smoking-asthma associations were analyzed with logistic regression. Additionally, restricted cubic splines were used to assess the nonlinear relationships between smoking characteristics and asthma incidence.

**RESULTS:**

Smokers had a 65% higher risk of incident asthma compared to non-smokers in middle-aged and older adults (HR=1.65; 95% CI: 1.10–2.46, p=0.015). According to stratified analysis, individuals with smoking duration ≥40 years (HR=1.95; 95% CI: 1.2–3.15, p=0.007), cumulative pack-years under 15 pack-years (HR=1.76; 95% CI: 1.04–2.99, p=0.035), and smoking onset before the age of 18 years (HR=2.31; 95% CI: 1.35–3.96, p=0.002) were at significantly greater risk for asthma. After controlling for cumulative pack-years, early smoking initiation (<18 years) remained an independent and significant predictor of asthma onset in middle and older age (HR=2.56; 95% CI: 1.29–5.06, p=0.007). Subgroup analysis revealed that smoking-related asthma risk was especially elevated among those aged <65 years, females, overweight individuals, and those without baseline comorbidities. Moreover, there was no significant difference in all-cause mortality between the smoking and non-smoking groups in asthma patients.

**CONCLUSIONS:**

The increased risk of asthma onset among middle-aged and older adults due to adolescent smoking initiation was independent of cumulative smoking pack-years, even though low pack-years and long-term smoking also contribute to increased risk. Targeted smoking cessation programs, especially adolescent prevention, are crucial to reduce asthma burden in this population.

## INTRODUCTION

Asthma is a common chronic airway disease that threatens public health^1^. Epidemiological evidence shows that asthma currently affects around 300 million individuals worldwide. In China, the cumulative prevalence of asthma among those aged ≥20 years has reached 4.2%, with a pronounced rise to >6.0% among the elderly (≥60 years)^2^. The accelerating pace of population aging, with those aged ≥65 years expected to comprise 20% of the global population by 2050^3^, presents a significant public health challenge in elderly asthma care. This stems from two main issues: diminished responsiveness to glucocorticoids and the frequent presence of multiple comorbid conditions, both of which complicate treatment and disease control^4^. This situation calls for a comprehensive investigation into the specific risk factors and modifiable intervention targets associated with asthma in the aging population.

Smoking, a modifiable behavioral factor, is of particular interest among many potential risks. There is substantial evidence that smoking is closely associated with increased risks of several diseases, including chronic bronchitis, lung cancer, and cardiovascular disease^5^. However, research on the impact of smoking on asthma in middle-aged and older adults has progressed relatively slowly, partly because this population is often excluded from most epidemiological studies and large-scale clinical trials due to concerns about potential comorbidity with chronic obstructive pulmonary disease (COPD)^6,7^. Even when such individuals are included, the results frequently fail to adequately adjust for confounding effects of COPD^8,9^.

The age at smoking initiation has varied considerably across different time periods, with >80% of adult smokers reporting having started smoking before the age of 18 years prior to 2012^10^. Lung development extends into adolescence, with most lung function established during adolescence. Smoking during adolescence has been identified as a key factor contributing to significant declines in lung function and the onset of respiratory symptoms^11^. Nevertheless, it remains unclear whether smoking initiation during adolescence independently contributes to the risk of asthma onset in later adulthood, beyond the effects of cumulative lifetime tobacco exposure.

The association between smoking and adult-onset asthma shows substantial heterogeneity across studies. In terms of sex differences, findings from the nationwide Canadian study indicate a significantly increased risk of asthma only among female smokers^8^. In contrast, a 10-year prospective study in Japan reported a 47% increased risk only among male smokers^9^. At the same time, some investigations have failed to identify a statistically significant link between smoking and asthma onset^12^. This inconsistency may be due to differences in study design (cross-sectional vs prospective), insufficient quantification of exposure parameters (missing key indicators such as age of smoking initiation, exposure duration, and smoking index), and selection bias due to the lack of longitudinal cohort data in middle-aged and elderly people^13^. Therefore, it is necessary to establish a standardized framework for assessing smoking exposure and to conduct long-term follow-up studies in middle-aged and older populations to better elucidate the true impact of smoking on asthma risk in this demographic.

Given the rapid aging of the world’s population, this study utilizes nationally representative data from the China Health and Retirement Longitudinal Study (CHARLS) to systematically examine the longitudinal associations between smoking behaviors, including smoking status, duration, pack-years, and age of initiation, with the incidence and all-cause mortality of asthma among middle-aged and older adults. By establishing a different smoking patterns exposure assessment model, this research fills critical evidence gaps and offers a foundation for precision prevention and management strategies targeting asthma in middle-aged and older populations.

## METHODS

### Study population

CHARLS is a nationally representative, large-scale prospective cohort study that employed a multi-stage stratified probability-proportional-to-size sampling method to recruit a representative sample of middle-aged and older community-dwelling residents across China^14^. The study was designed to address key issues of population aging^14^. Ethical approval was obtained from the Institutional Review Board of Peking University (IRB00001052–11015).

**Figure 1:**
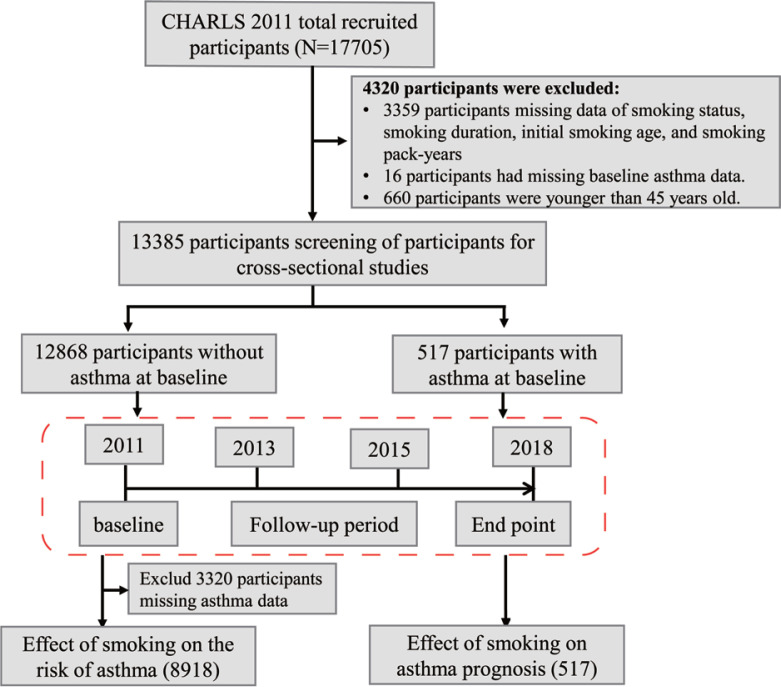
The flow chart of the study

Based on the 2011 baseline survey of CHARLS (n=17705), this study included community-dwelling adults aged ≥45 years. We excluded 3599 individuals with missing data on smoking-related variables (smoking status, pack-years, age at smoking initiation), 61 participants with incomplete asthma diagnosis information, and 660 participants aged <45 years. After exclusions, 13385 participants were eligible for the cross-sectional analysis of smoking exposure and asthma (Figure 1). For longitudinal analysis, two independent cohorts were constructed: 1) Incident asthma cohort. From the baseline population (n=13385), we excluded 517 with baseline asthma and 3950 individuals with missing asthma status during follow-up, resulting in 8918 asthma-free participants who completed four waves of follow-up (2011–2018) to evaluate the association between smoking exposure and incident asthma; and 2) Asthma prognosis cohort. A total of 517 participants with physician-diagnosed asthma at baseline and complete mortality records were included to investigate the impact of smoking behavior on all-cause mortality among asthma patients (Figure 1).

### Assessment of asthma

In the CHARLS study, asthma outcomes were identified through self-reported data. Structured in-home interviews were conducted by uniformly trained interviewers, and asthma diagnosis was determined based on participants’ responses to the question: ‘Has a doctor ever diagnosed you with asthma?’.

### Mortality outcome assessment

All-cause mortality was determined through participant survival status verification (alive/deceased) across Waves 2–4. For individuals who survived the observation period, the survival time was calculated as the interval between Wave 1 and the last follow-up interview. For those who experienced a mortality event, the survival time was calculated as the median of the two follow-up waves: the wave with recorded death information and its preceding wave. Given substantial missing data on specific mortality causes, we employed all-cause mortality as the study endpoint.

### Assessment of smoking status

Smoking status was assessed based on baseline interview data and categorized into non-smokers and smokers, with smokers including both current and former smokers. Among smokers, detailed information was extracted regarding smoking duration, daily cigarette consumption, age at starting smoking, and cumulative pack-years of smoking. Smoking duration was categorized into three groups: <20, 20–39, and ≥40 years. Age at starting smoking was classified into three groups: <18, 18 to <25, ≥25 years. Based on this information, pack-years of smoking were calculated as smoking duration (years) multiplied by the average daily cigarette consumption, then divided by 20. Pack-years of smoking were classified into three groups: <15, 15 to <30, ≥30.

### Covariates

Covariates were assessed based on baseline interview data. The following potential confounders were considered: age, sex (female, male), body mass index (BMI), education level (primary school, high school), marital status (married, non-married), residence (rural, urban), alcohol drinking (yes, no), hypertension (yes, no), cardiovascular disease (CVD) (yes, no), and COPD (yes, no). Hypertension was determined based on a self-reported history of hypertension. COPD was determined based on self-reported chronic respiratory conditions, including chronic bronchitis, emphysema, or pulmonary heart disease, excluding tumors or cancer^15^.

### Statistical analysis

Baseline characteristics of participants were described according to smoking status. Continuous variables with a non-normal distribution are presented as the median and interquartile range (IQR), while those with a normal distribution as mean ± standard deviation (SD). Categorical variables are reported as frequencies and percentages. Group differences were assessed using t-tests for normally distributed variables, non-parametric tests for skewed distributions, and chi-squared tests for categorical data. The proportion of missing values for key covariates is provided in Supplementary file Table S1. Missing data for covariates were handled using multiple imputation via chained equations (MICE) in the R package *mice*. Five imputations were performed using the predictive mean matching method, with a maximum of 50 iterations per dataset. The first completed dataset was selected for the final analysis.

In the cross-sectional analysis, multivariable logistic regression models were used, yielding odds ratios (ORs) and corresponding 95% confidence intervals (CIs). Using longitudinal data from 2011 to 2018, Nelson-Aalen cumulative hazard curves and Kaplan-Meier survival curve illustrated asthma incidence and all-cause mortality across different smoking behaviors, with group differences assessed by the log-rank test. This association was further examined using Cox proportional hazards regression. Three regression models were constructed using non-smokers as the reference category. Model 1 was unadjusted, with smoking factor as the only variable. Model 2 accounted for demographic confounders and health conditions, including age, sex, BMI, education level, marital status, alcohol drinking, hypertension and CVD. Model 3 was adjusted as for Model 2 plus COPD. To explore the differential effects of smoking on asthma onset, we performed a series of subgroup analyses based on covariates. To determine the extent to which sociodemographic characteristics and health-related behaviors influence the association between smoking status and asthma, interaction analysis was conducted based on the product terms [(smoking status) × (interaction term)] from the primary analysis. To test the proportional hazards assumption, we applied Schoenfeld residuals analysis using the *cox.zph* function in R, as presented in Supplementary file Tables S2 and S3.

Additionally, restricted cubic splines (RCS) were used to examine potential nonlinear associations and visualize the relationships between smoking exposures and the risk of asthma onset. We selected five knots for smoking pack-years and three knots for smoking duration, in line with common practice in the literature, which typically recommends using three to five knots to balance model flexibility and the risk of overfitting^16^. The significance of the nonlinear spline components was tested jointly using the Wald test. We applied the chi-squared test and pairwise proportion tests to compare asthma prevalence and incidence by age at smoking initiation. Cumulative pack-years were analyzed using the Kruskal-Wallis test with Dunn’s test for *post hoc* comparisons. Data analysis was conducted using R software (version 4.2.2) and GraphPad Prism 7.0 software (GraphPad, San Diego, CA, USA). All tests were two-sided, and a p<0.05 was considered statistically significant.

### Sensitivity analysis

To evaluate the robustness of our findings, two sensitivity analyses were conducted. First, due to the high proportion of missing values for BMI, we repeated primary regression models after excluding subjects with missing BMI values. Second, given potential confounding effect of COPD, we excluded participants with both asthma and COPD (i.e. asthma–COPD overlap, ACO) and repeated the primary analyses.

## RESULTS

### Baseline characteristics

Table 1 summarizes baseline characteristics of 13385 participants, including 517 with asthma. The median age was 57 years (IQR: 51–64), and 39.2% of the participants were male. Compared to those without asthma, individuals with asthma were generally older, more likely to be male, less educated, and more frequently single, divorced, or widowed. They also had higher rates of comorbidities (hypertension, CVD, and COPD) and greater tobacco exposure, including higher smoking prevalence, longer smoking duration, and more pack-years.

**Table 1:** Characteristics of participants according to asthma status at baseline, using China Health and Retirement Longitudinal Study (CHARLS) data

*Characteristics*	*Overall* *n (%)*	*Asthma* *n (%)*	*non-Asthma* *n (%)*	p*
**Total**, n	13385	517	12868	
**Age** (years), median (IQR)	57.0 (51.0–64.0)	61.0 (54.0–69.0)	57.0 (51.0–64.0)	<0.001
**Sex**				0.001
Female	8134 (60.8)	277 (53.6)	7857 (61.1)	
Male	5251 (39.2)	240 (46.4)	5011 (38.9)	
**BMI** (kg/m^2^), median (IQR)	23.2 (20.9–25.9)	23.0 (20.2–26.2)	23.2 (20.9–25.9)	0.137
**Residence**				0.711
City/town	3104 (23.2)	116 (22.4)	2988 (23.2)	
Village	10271 (76.8)	401 (77.6)	9870 (76.8)	
**Alcohol drinking**				0.145
No	8938 (66.8)	329 (63.8)	8609 (66.9)	
Yes	4439 (33.2)	187 (36.2)	4252 (33.1)	
**Education level**				<0.001
Elementary school or lower	8908 (66.6)	387 (75.0)	8521 (66.2)	
Middle school or higher	4474 (33.4)	129 (25.0)	4345 (33.8)	
**Marital status**				<0.001
Married	11665 (87.2)	414 (80.2)	11251 (87.4)	
Single/divorced/widowed	1719 (12.8)	102 (19.8)	1617 (12.6)	
**Smoking status**				0.001
Non-smoker	9985 (74.6)	352 (68.1)	9633 (74.9)	
Smoker (current or ever)	3400 (25.4)	165 (31.9)	3235 (25.1)	
**Smoking duration** (years), median (IQR)	0.0 (0.0–5.0)	0.0 (0.0–31.0)	0.0 (0.0–2.0)	<0.001
**Smoking duration** (years)				<0.001
0	9985 (74.6)	352 (68.1)	9633 (74.9)	
<20	280 (2.1)	9 (1.7)	271 (2.1)	
20–39	1986 (14.8)	75 (14.5)	1911 (14.9)	
≥40	1134 (8.5)	81 (15.7)	1053 (8.2)	
**Cigarettes smoked/day**, median (IQR)	0.0 (0.0–1.0)	0.0 (0.0–10.0)	0.0 (0.0–1.0)	0.001
**Cigarettes smoked/day**				0.001
0	9985 (74.6)	352 (68.1)	9633 (74.9)	
<20	1354 (10.1)	76 (14.7)	1278 (9.9)	
20–39	1695 (12.7)	73 (14.1)	1622 (12.6)	
≥40	351 (2.6)	16 (3.1)	335 (2.6)	
**Smoking pack-years**, median (IQR)	0.0 (0.0–1.4)	0.0 (0.0–18.5)	0.0 (0.0–0.6)	<0.001
**Smoking pack-years**				<0.001
0	9985 (74.6)	352 (68.1)	9633 (74.9)	
<15	798 (6.0)	26 (5.0)	772 (6.0)	
15–30	965 (7.2)	53 (10.3)	912 (7.1)	
≥30	1637 (12.2)	86 (16.6)	1551 (12.1)	
**Age started smoking** (years), median (IQR)	20.0 (18.0–25.0)	20.0 (18.0–25.0)	20.0 (16.0–25.0)	0.003
**Age started smoking** (years)				<0.001
<18	782 (5.8)	54 (10.4)	728 (5.7)	
18–25	1566 (11.7)	68 (13.2)	1498 (11.6)	
≥25	1052 (7.9)	43 (8.3)	1009 (7.8)	
**Comorbidities**				
Hypertension	5272 (45.9)	248 (53.0)	5024 (45.6)	0.002
CVD	1616 (12.1)	136 (26.7)	1480 (11.6)	<0.001
COPD	1173 (8.8)	316 (61.4)	857 (6.7)	<0.001

BMI: body mass index. CVD: cardiovascular disease. COPD: chronic obstructive pulmonary disease. IQR: interquartile range.

* Chi-squared test or Fisher’s exact test.

### The association between smoking behaviors and asthma prevalence

The cross-sectional findings on the relationship between smoking behaviors and asthma prevalence are shown in Table 2. Smokers had a significantly higher prevalence of asthma compared to non-smokers (OR=1.40; 95% CI: 1.15–1.68, p=0.001). Stratified analyses revealed that individuals with long-term smoking (≥40 years), high cumulative exposure (≥15 pack-years), and those who initiated smoking aged <18 years were at particularly higher risk of asthma than non-smokers. These associations remained significant after adjusting for demographic and health-related factors (Model 2). However, after further adjusting for COPD (Model 3), only early smoking initiation (<18 years) remained significantly associated with asthma (AOR=1.50; 95% CI: 1.03–2.17, p=0.033).

**Table 2:** Association between smoking behaviors and asthma prevalence in middle-aged and older adults, within the China Health and Retirement Longitudinal Study (CHARLS) study

*Variables*	*Model 1*		*Model 2*		*Model 3*	
	*OR (95% CI)*	p*	*AOR (95% CI)*	p*	*AOR (95% CI)*	p*
**Smoking status**						
Non-smoker ®						
Smoker	1.4 (1.15–1.68)	**0.001**	1.31 (1.03–1.68)	**0.031**	1.17 (0.90–1.54)	0.241
**Smoking duration**	1.01 (1.01–1.02)	**<0.001**	1.01 (1.00–1.02)	**0.001**	1.01 (1.00–1.01)	**0.044**
**Smoking duration by group**						
0 ®						
<20	0.91 (0.43–1.68)	0.781	0.99 (0.46–1.86)	0.973	0.89 (0.40–1.77)	0.757
20–39	1.07 (0.83–1.38)	0.582	1.19(0.87–1.61)	0.278	1.22 (0.87–1.71)	0.249
≥40	2.11 (1.63–2.69)	**<0.001**	1.52 (1.12–2.06)	**0.006**	1.19 (0.86–1.66)	0.291
**Pack-years**	1.01 (1.00–1.01)	<0.001	1.01 (1.00–1.01)	0.024	1 (1.00–1.01)	0.195
**Pack-years by category**						
0 ®						
<15	0.92 (0.60–1.35)	0.693	0.87 (0.56–1.32)	0.532	0.8 (0.50–1.24)	0.334
15–30	1.59 (1.17–2.12)	**0.002**	1.64 (1.15–2.29)	**0.005**	1.46 (1.00–2.11)	**0.049**
≥30	1.52 (1.18–1.92)	**0.001**	1.4 (1.04–1.88)	**0.027**	1.24 (0.90–1.72)	0.189
**Age started smoking by group**						
non-smokers ®						
<18	2.03 (1.49–2.71)	**<0.001**	1.85 (1.30–2.58)	**<0.001**	1.5 (1.03–2.17)	**0.033**
18–25	1.24 (0.95–1.61)	0.109	1.23 (0.89–1.69)	0.196	1.1 (0.78–1.55)	0.585
≥25	1.17 (0.83–1.59)	0.351	1.07 (0.74–1.51)	0.724	1.02 (0.69–1.49)	0.911

Model 1: unadjusted model. Model 2: adjusted for age, sex, BMI, education level, marital status, alcohol drinking, hypertension and CVD. Model 3: adjusted as for Model 2 plus COPD. AOR: adjusted odds ratio. ® Reference categories.

* Statistically significant at p<0.05.

### The association between smoking behaviors and asthma incidence

**Figure 2:**
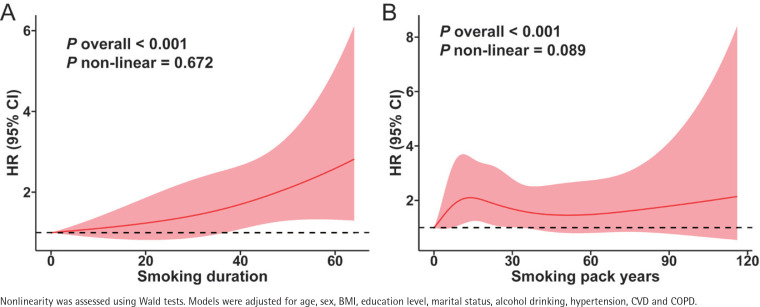
RCS-based Cox regression plots showing the association between smoking exposure with asthma risk in middle-aged and older adults: A) Smoking duration with asthma onset; B) Smoking pack-years with asthma onset. Red lines show adjusted hazard ratios and shaded areas indicate 95% confidence intervals

During the 2011–2018 follow-up period, 214 new-onset asthma cases (2.4%) occurred in 8918 asthma-free individuals at baseline. Nelson-Aalen curves indicated a higher cumulative hazard of asthma among smokers, especially in those with longer smoking duration (≥40 years) and those who started smoking before the age of 18 years, both of which showed statistically significant group differences (Supplementary file Figure S1 A–D). In the fully adjusted Cox regression model (Table 3), smoking was associated with a 65% higher risk of asthma compared to non-smoking (HR=1.65; 95% CI: 1.10–2.46, p=0.015). Stratified analyses revealed that individuals who smoked for ≥40 years (HR=1.95; 95% CI: 1.20–3.15, p=0.007), those with <15 pack-years of cumulative exposure (HR=1.76; 95% CI: 1.04–2.99, p=0.035), and those who started smoking before the age of 18 years (HR=2.31; 95% CI: 1.35–3.96, p=0.002) were all at significantly increased risk of developing asthma. Restricted cubic spline (RCS) analyses showed significant associations of both smoking pack-years and duration with asthma risk (overall p<0.001). However, as both p-values for nonlinearity exceeded 0.05, these data indicated no apparent nonlinear relationships (Figure 2).

**Table 3:** Cox proportional hazard ratios for the association between smoking behaviors and asthma incidence over a 7-year follow-up in middle-aged and older adults using 2011-2018 China Health and Retirement Longitudinal Study (CHARLS) data

*Variables*	*Model 1*		*Model 2*		*Model 3*	
	*HR (95% CI)*	p*	*AHR (95% CI)*	p*	*AHR (95% CI)*	p*
**Smoking status**						
Non-smoker ®						
Smoker	1.4 (1.05–1.87)	**0.02**	1.88 (1.26–2.81)	**0.002**	1.65 (1.10–2.46)	**0.015**
**Smoking duration**	1.01 (1.01–1.02)	**<0.001**	1.02 (1.01–1.03)	**<0.001**	1.01 (1.00–1.02)	**0.003**
**Smoking duration by group**						
0 ®						
<20	1.17 (0.48–2.85)	0.73	1.61 (0.65–4.00)	0.309	1.36 (0.55–3.38)	0.503
20–39	0.94 (0.63–1.41)	0.767	1.5 (0.91–2.49)	0.112	1.46 (0.88–2.42)	0.145
≥40	2.38 (1.65–3.43)	**<0.001**	2.44 (1.52–3.92)	**<0.001**	1.95 (1.20–3.15)	**0.007**
p for trend	**0.001**		**<0.001**		**0.007**	
**Pack-years**	1.01 (1.00–1.01)	0.069	1.01 (1.00–1.02)	0.047	1.01 (1.00–1.01)	0.144
**Pack-years by category**						
0 ®						
<15	1.63 (1.01–2.62)	**0.046**	2 (1.18–3.39)	**0.01**	1.76 (1.04–2.99)	**0.035**
15–30	1.3 (0.80–2.12)	0.292	1.88 (1.07–3.30)	**0.029**	1.7 (0.96–2.99)	0.067
≥30	1.35 (0.92–1.98)	0.121	1.79 (1.10–2.93)	**0.02**	1.52(0.93–2.50)	0.098
p for trend	0.058		**0.015**		0.078	
**Age started smoking by group**						
Non-smoker ®						
<18	2.09 (1.33–3.27)	**0.001**	2.81 (1.64–4.80)	**<0.001**	2.31 (1.35–3.96)	**0.002**
18–25	1.08 (0.71–1.66)	0.708	1.6 (0.95–2.71)	0.08	1.41 (0.83–2.39)	0.201
≥25	1.41 (0.90–2.21)	0.132	1.68 (1.01–2.79)	**0.045**	1.51 (0.91–2.51)	0.114

Model 1: unadjusted model. Model 2: adjusted for age, sex, BMI, education level, marital status, alcohol drinking, hypertension and CVD. Model 3: adjusted as for Model 2 plus COPD. AHR: adjusted hazard ratio. ® Reference categories.

* Statistically significant at p<0.05.

**Figure 3:**
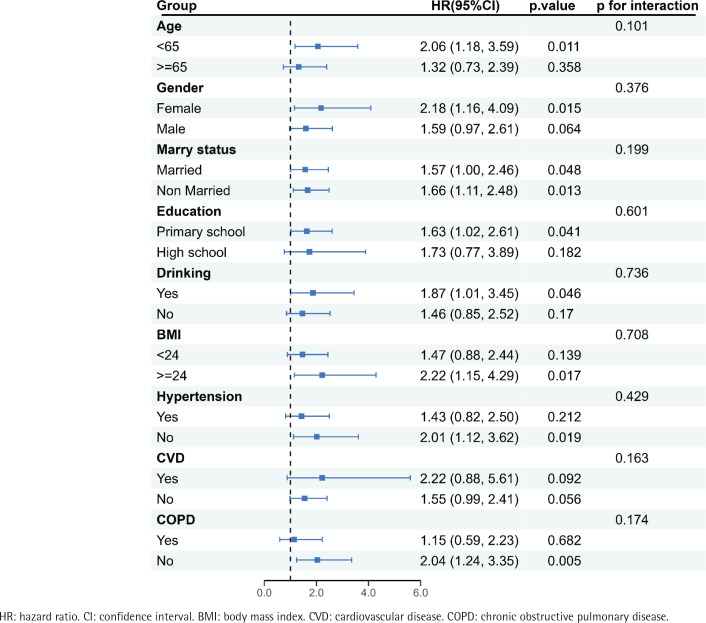
Forest plot of subgroup analysis of the association of smoking status with the risk of new-onset asthma

Subgroup analyses revealed that the association between smoking and asthma risk was significant in individuals aged <65 years, females, alcohol consumers, those with BMI ≥24 kg/m^2^, and those without hypertension or COPD (Figure 3). No significant associations were observed in other subgroups. Moreover, no statistically significant interactions between smoking and any of these covariates (p for interaction >0.05) were observed.

### Smoking before the age of 18 years as an independent risk factor for asthma incidence

**Figure 4:**
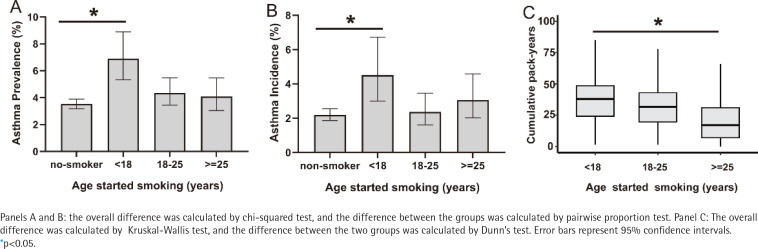
Associations between age of smoking initiation with asthma outcomes and cumulative smoking pack-years: A and B) Prevalence and incidence of asthma by age started smoking; C) Cumulative pack-years by age started smoking

In order to explore the relationship between early-life smoking and asthma onset in later life, we conducted an analysis based on the age of smoking initiation. Individuals who started smoking before the age of 18 years showed significantly higher asthma prevalence and incidence compared to non-smokers (Figures 4A and 4B). This early-smoking group also had greater cumulative tobacco exposure (pack-years) (Figure 4C). In order to assess whether early initiation of smoking independently contributes to asthma onset, we extended the multivariable Cox proportional hazards model (Model 3) by adjusting for cumulative smoking exposure (pack-years). Even after controlling for cumulative pack-years, early smoking initiation (<18 years) remained an independent and significant predictor of asthma onset in middle-aged and older adults (HR=2.56; 95% CI: 1.29–5.06, p=0.007).

### The relationship between smoking behaviors and all-cause mortality in asthma patients

Among 517 asthma patients followed longitudinally, smoking was not significantly associated with increased all-cause mortality, regardless of adjustment for potential confounders (Table 4). Although unadjusted analyses suggested elevated mortality risks among long-term smokers (≥40 years; HR=2.07; 95% CI: 1.29–3.31, p=0.003) and those who began smoking before the age of 18 years (HR=1.79; 95% CI: 1.02–3.17, p=0.044), these associations did not remain statistically significant after multivariable adjustment (Model 3). Kaplan-Meier analysis also supported the lack of a significant survival difference between smokers and non-smokers with asthma (Supplementary file Figure S1 E). Overall, this study did not observe a significant impact of smoking on mortality risk among asthma patients.

**Table 4:** Hazard ratios for all-cause mortality comparing smokers and non-smokers with asthma patients over a 7-year follow-up using 2011–2018 China Health and Retirement Longitudinal Study (CHARLS) data

*Variables*	*Model 1*		*Model 2*		*Model 3*	
	*HR (95% CI)*	p*	*AHR (95% CI)*	p*	*AHR (95% CI)*	p*
**Smoking status**						
Non-smoker ®						
Smoker	1.22 (0.79–1.88)	0.375	1.22 (0.74–2.01)	0.436	1.23 (0.75–2.04)	0.41
**Smoking duration**	1.01 (1.00–1.02)	**0.01**	1.01 (1.00–1.02)	0.238	1.01 (1.00–1.02)	0.219
**Smoking duration by group**						
0 ®						
<20	0.68 (0.09–4.88)	0.697	0.94 (0.13–6.89)	0.951	0.95 (0.13–6.95)	0.957
20–39	0.47 (0.20–1.09)	0.08	0.88 (0.34–2.26)	0.79	0.89 (0.35–2.31)	0.819
≥40	2.07 (1.29–3.31)	**0.003**	1.33 (0.79–2.25)	0.286	1.34 (0.79–2.28)	0.271
p for trend	0.065		0.33		0.31	
**Pack-years**	1.01 (1.00–1.02)	0.095	1.01 (1.00–1.01)	0.263	1.01 (1.00–1.01)	0.253
**Pack-years by category**						
0 ®						
<15	0.71 (0.22–2.28)	0.569	0.72 (0.22–2.31)	0.577	0.72 (0.22–2.33)	0.585
15–30	1.45 (0.78–2.70)	0.242	1.76 (0.85–3.64)	0.128	1.77 (0.86–3.67)	0.123
≥30	1.23 (0.72–2.11)	0.455	1.2 (0.66–2.20)	0.549	1.22 (0.67–2.24)	0.517
p for trend	0.301		0.4		0.373	
**Age started smoking by group**						
Non-smoker ®						
<18	1.79 (1.02–3.17)	**0.044**	1.71 (0.93–3.14)	0.084	1.74 (0.95–3.22)	0.075
18–25	1.1 (0.59–2.05)	0.764	1.22 (0.61–2.46)	0.571	1.23 (0.61–2.48)	0.558
≥25	0.71 (0.28–1.77)	0.464	0.62 (0.24–1.63)	0.336	0.63 (0.24–1.65)	0.35

Model 1: unadjusted model. Model 2: adjusted for age, sex, BMI, education level, marital status, alcohol drinking, hypertension and CVD. Model 3: adjusted as for Model 2 plus COPD. AHR: adjusted hazard ratio. ® Reference categories.

* Statistically significant at p<0.05.

### Sensitivity analysis

Sensitivity analyses excluding participants with missing BMI values produced results consistent with the primary models (Supplementary file Table S4). In addition, excluding those who developed ACO during follow-up left only 65 pure asthma cases out of 214, as 149 (69.6%) were classified as ACO. The resulting sample size reduction limited statistical power, but the direction of associations remained consistent with the main analysis, supporting the robustness of our findings (Supplementary file Table S5).

## DISCUSSION

Based on a large-scale cohort of middle-aged and older adults in China, this study investigated the association between smoking behaviors and the risk of asthma onset and all-cause mortality, with adjustment for comorbidities, including COPD. Smoking increases the risk of asthma onset in middle-aged and older adults; however, it does not appear to influence overall mortality rates. Stratified analysis revealed that long-term smokers, those with low cumulative exposure, and those smoked before the age of 18 years had a particularly elevated risk of asthma incidence. Notably, the increased risk of asthma onset due to adolescent smoking initiation was independent of cumulative smoking pack-years. This finding provides valuable longitudinal evidence on how different smoking characteristics influence asthma incidence and all-cause mortality, thereby enhancing our understanding of smoking’s role in asthma pathogenesis, and emphasizing the critical need for early interventions in high-risk smoking populations, especially among adolescent smokers.

An association between smoking and asthma prevalence was identified in cross-sectional analyses; however, this association was substantially reduced after controlling for COPD, suggesting the confounding effect of COPD. Smoking facilitates the onset of COPD by triggering oxidative stress and persistent inflammation^17^. There is a clinical overlap between COPD and asthma, particularly in older adults^18^, failing to account for COPD may overestimate the effect of smoking on asthma prevalence. Statistical adjustment for COPD helps mitigate this bias and yields a more accurate estimate of the true effect of smoking on asthma.

Based on a 7-year longitudinal cohort study, smoking was found to significantly increase the risk of incident asthma among middle-aged and older adults after adjusting for various covariates, including COPD. Our findings reinforce the association between smoking and asthma onset, highlighting its possible pathogenic role. The longitudinal design allowed for clearer identification of smoking as an independent risk factor, reducing bias commonly encountered in cross-sectional research.

The relationship between smoking and asthma risk is complex and may vary depending on smoking intensity and contributing factors. Whereas previous research indicated a positive dose–response relationship between smoking and asthma risk^9,19^, our study found that in middle-aged and older individuals, only lower cumulative smoking levels were significantly linked to asthma incidence. Our finding aligns with a prior investigation conducted in a population aged 21–63 years that excluded individuals with COPD^20^. Because smoking is a definitive risk factor for COPD, the inclusion of COPD patients in asthma analyses could distort results by overstating their association with smoking. Experimental data from animal studies indicate that cigarette smoke exposure downregulates antigen-specific T cell function in a dose-dependent manner, suppressing allergic airway inflammation. Low-dose exposure appears to facilitate allergic sensitization and asthma onset, while high-dose exposure inhibits local allergic responses but induces systemic inflammation, potentially accelerating the development of COPD and other irreversible obstructive lung diseases^21^. These mechanisms may partially explain why only lower cumulative smoking levels were associated with an increased risk of asthma in the older population studied.

Stratified analysis by smoking duration indicated that individuals with ≥40 years of smoking history had a significantly increased risk of asthma, with a statistically significant trend p-value. This finding suggests a duration–response relationship, whereby prolonged smoking is associated with a higher risk of asthma. A plausible explanation is that prolonged smoking induces a series of structural and functional alterations in the airways, including epithelial injury, airway remodeling, excessive mucus production, and increased airway responsiveness^22^. These pathological changes compromise airway integrity and immune regulation, thereby heightening the susceptibility to asthma in older adults.

This study demonstrates that initiating smoking before the age of 18 years independently and significantly increases the risk of developing asthma among middle-aged and older adults, a novel finding of our research. The results suggest that early smoking behavior itself is an independent risk factor for asthma onset in middle-aged and older adults, and its effect potentially does not entirely depend on the cumulative degree of tobacco exposure. Previous prospective studies in adolescents have shown that smoking during adolescence increases the risk of asthma by two- to three-fold^23,24^. However, these researches have focused on youth or young adults, and evidence regarding the long-term impact of early smoking initiation on asthma risk in middle-aged and older populations remains limited. Our findings fill this critical gap by providing longitudinal evidence that early tobacco exposure has lasting detrimental effects on lung health. The adolescent stage is a pivotal phase of lung maturation, where the airways are especially vulnerable to environmental exposures, including tobacco smoke. Smoking during this sensitive period may compromise lung reserve and elevate the likelihood of developing chronic respiratory conditions later in life^25^. Animal research has provided evidence that nicotine exposure during this critical developmental window of adolescence compromises alveolar growth and leads to permanent lung function decline^26^. In summary, initiating smoking during adolescence is not merely a short-term behavioral risk but a determinant with long-term health consequences.

Given the potential confounding effect of COPD, we performed a sensitivity analysis excluding participants with ACO and repeated the primary analyses. ACO accounted for approximately 70% of asthma cases in our cohort, consistent with prior findings in middle-aged and older adults^2,27^. Although the reduced sample size limited statistical power and yielded non-significant associations, the effect estimates remained directionally consistent with the primary analysis, supporting its robustness. Furthermore, smoking modifies the asthma phenotype, often leading to fixed airflow limitation in asthma patients^28^. Excluding ACO may lead to an underestimation of the true association between smoking and asthma incidence in this age group. Therefore, we retained ACO cases in the main analysis and adjusted for baseline COPD to minimize confounding while preserving power and generalizability.

Sex-stratified analysis revealed that the association between smoking and asthma onset was significantly stronger in women than in men, suggesting a sex-based difference in susceptibility. This aligns with findings from the Canadian National Population Health Survey, where smoking predominantly increased asthma risk among women^8^. Based on existing evidence, we hypothesize that this sex difference may be attributed to: 1) women’s increased vulnerability to tobacco smoke-induced oxidative stress in the airways^29^; and 2) cigarette smoke exacerbating the influence of female hormones-particularly progesterone-on allergic asthma phenotypes, thereby elevating IgE levels^30^. Additionally, the association between smoking and incident asthma in middle-aged and older adults was particularly evident among individuals with obesity. Age-related declines in lung function, compounded by smoking-induced airway inflammation, may elevate asthma susceptibility in older adults^31^. Obesity further contributes to systemic low-grade inflammation, especially via visceral fat accumulation and metabolic dysfunction, leading to increased levels of IL-6, C-reactive protein, and tumor necrosis factor-α (TNF-α), which can trigger or worsen airway hyperresponsiveness^32^. Notably, obesity-related asthma is often characterized by a neutrophilic inflammatory phenotype, which is strongly linked to smoking and typically responds poorly to standard treatment^33^.

Smoking is associated with neutrophilic airway inflammation and may reduce eosinophil sensitivity to systemic corticosteroids. Continuous exposure to cigarette smoke leads to a more severe asthma phenotype and inadequate asthma control^34^. In a 7-year follow-up of 517 asthma patients, we observed no significant difference in all-cause mortality between smokers and non-smokers after adjusting for multiple variables. This could be attributed to the limited sample size and relatively short follow-up, which may have resulted in inadequate statistical power to detect modest mortality differences between smokers and non-smokers. Larger prospective studies with extended follow-up are needed to clarify the long-term effects of smoking on asthma-related mortality.

### Strengths and limitations

This is a large-scale cohort study that explores how smoking parameters affect the incidence and mortality risk of asthma in middle-aged and older adults, using dose-response analysis and nonlinear testing. These findings contribute to the understanding of smoking’s role in the pathogenesis of asthma and underscore the importance of early intervention in high-risk smoking populations, especially among adolescent smokers.

However, this study also has some limitations. First, asthma diagnosis was primarily based on self-reported physician diagnosis without information on objective clinical verification such as spirometry, standardized diagnostic criteria, or physician specialty. This may lead to reporting bias or misclassification bias, especially when there is high clinical overlap between asthma and COPD in the elderly population. Secondly, smoking information was self-reported, which may introduce recall bias. Third, as an observational study, it cannot establish a causal relationship between smoking and asthma risk. Finally, because the study sample consisted of middle-aged and older adults from China, caution is warranted when extrapolating these findings to younger individuals or populations in different countries or ethnic backgrounds. Future studies should consider longitudinal designs incorporating more objective diagnostic criteria and comprehensive data collection to better explore the underlying mechanisms linking smoking and asthma, while more effectively controlling for confounding factors, thereby enhancing understanding in this field.

## CONCLUSIONS

The increased risk of asthma onset among middle-aged and older adults due to adolescent smoking initiation was independent of cumulative smoking pack-years, even though low pack-years and long-term smoking also contribute to increased risk. Targeted smoking cessation programs, especially adolescent prevention, may be crucial to reduce asthma burden in this population.

## Supplementary Material



## Data Availability

Data sharing is not applicable to this article as no new data were created.
