# Supplementary material for: Impact of smoking behaviors on asthma incidence and allcause mortality in middle-aged and older adults: A longitudinal study from China
Source: Tob Induc Dis. 2025 Sep 9;23:10.18332/tid/207912. doi: 10.18332/tid/207912 (PMC12418942; doi:10.18332/tid/207912)
Supplement: Supplementary file 1 [file TID-23-126-s1.pdf]

Supplementary materials

Table S1. Proportion of missing data for covariates

**Covariates**	**Missing counts**	**Percent (%)**
BMI	2742	20.49
Drinking	8	0.06
Education group	3	0.02
Marriage group	1	0.01
Hypertension	1901	14.20
CVD	67	0.50
COPD	49	0.37

Abbreviation: BMI, body mass index; CVD, cardiovascular disease; COPD, chronic obstructive pulmonary disease

Table S2. Proportional Hazards Assumption Test Results for Cox Models of Smoking and Asthma Incidence

Variables	Model 1		Model 2		Model 3	
	PH p.value	Global PH p.value	PH p.value	Global PH p.value	PH p.value	Global PH p.value
Smoking status	0.694	0.69	0.7	0.04	0.71	0.06
Smoking duration	0.62	0.62	0.65	0.05	0.67	0.07
Smoking duration group	0.59	0.59	0.59	0.07	0.6	0.1
Pack years	0.45	0.45	0.47	0.04	0.45	0.04
Pack year category	0.96	0.96	0.96	0.08	0.964	0.12
Age started smoking group	0.92	0.92	0.92	0.08	0.9	0.1

Table S3. Proportional Hazards Assumption Test Results for Cox Models of Smoking and Asthma all-cause mortality

Variables	Model 1		Model 2		Model 3	
	PH p.value	Global PH p.value	PH p.value	Global PH p.value	PH p.value	Global PH p.value
Smoking status	0.84	0.84	0.89	0.5	0.89	0.51
Smoking duration	0.5	0.5	0.56	0.44	0.56	0.43
Smoking duration group	0.94	0.94	0.95	0.65	0.95	0.64
Pack years	0.69	0.69	0.66	0.46	0.66	0.48
Pack year category	0.53	0.53	0.59	0.43	0.59	0.43
Age started smoking group	0.5	0.5	0.49	0.41	0.49	0.41

Table S4. Cox proportional hazard ratios for the association of smoking behaviors with asthma incidence over a 7-year follow-up period in middle aged and olde adults without BMI missing data.

Variable	**Model 1**		**Model 2**		**Model 3**	
	HR (95%CI)	*p*-value	HR (95%CI)	*p*-value	HR (95%CI)	*p*-value
Smoking status						
non-smoker	reference					
smoker	**1.45(1.06-1.97)**	**0.02**	**2.12(1.36-3.32)**	**<0.001**	**1.85(1.17-2.93)**	**0.008**
Smoking duration	**1.01(1.01-1.02)**	**<0.001**	**1.02(1.01-1.03)**	**<0.001**	**1.02(1.01-1.03)**	**0.002**
Smoking duration group						
0	reference					
<20	1.42(0.58-3.47)	0.444	1.7(0.61-4.70)	0.309	1.49(0.54-4.14)	0.441
20-39	0.95(0.62-1.48)	0.835	1.69(0.97-2.96)	0.064	1.65(0.93-2.92)	0.086
>=40	**2.42(1.63-3.60)**	**<0.001**	**2.82(1.66-4.78)**	**<0.001**	**2.18(1.27-3.75)**	**0.005**
p for trend	**0.002**		**<0.001**		**0.004**	
Pack years	**1.01(1.00-1.01)**	**0.045**	**1.01(1.00-1.02)**	**0.014**	1.01(1.00-1.02)	0.055
Pack year category						
0	reference					
<15	**1.74(1.05-2.90)**	**0.032**	**2.22(1.24-3.97)**	**0.007**	**1.93(1.07-3.47)**	**0.028**
15-30	1.19(0.69-2.08)	0.529	1.9(1.00-3.62)	0.05	1.75(0.91-3.35)	0.091
>=30	**1.45(0.97-2.18)**	**0.073**	**2.19(1.27-3.76)**	**0.005**	**1.85(1.06-3.21)**	**0.03**
p for trend	0.05		**0.005**		**0.033**	
Age started smoking group						
non-smoker	reference					
<18	**2.21(1.38-3.55)**	**0.001**	**3.43(1.92-6.15)**	**<0.001**	**2.7(1.50-4.87)**	**<0.001**
18-25	1.19(0.76-1.86)	0.451	1.99(1.12-3.54)	0.019	1.75(0.98-3.12)	0.059
>=25	1.3(0.78-2.17)	0.306	1.64(0.91-2.95)	0.1	1.48(0.82-2.70)	0.196

Model 1, unadjusted model; Model 2, adjusted for age, sex, BMI, education, marital status, drinking, hypertension and CVD; Model 3, adjusted for age, sex, BMI, education, marital status, drinking, hypertension, CVD and COPD. Abbreviation: HR, hazard ratio; CI, confidence intervals.

Table S5. Cox proportional hazard ratios for the association of smoking behaviors with asthma incidence over a 7-year follow-up period in middle aged and olde adults excluding subject with ACOS.

**Variable**	**Mode 1**		**Model 2**		**Model 3**	
	**HR (95%CI)**	***p*-value**	**HR (95%CI)**	***p*-value**	**HR (95%CI)**	***p*-value**
Smoking status						
non-smoker	reference					
smoker	0.97(0.55-1.70)	0.907	1.26(0.55-2.89)	0.582	1.31(0.57-2.97)	0.525
Smoking duration	1(0.99-1.02)	0.955	1.01(0.99-1.03)	0.381	1.01(0.99-1.03)	0.332
Smoking duration group						
0	reference					
<20	2.07(0.65-6.65)	0.22	1.83(0.42-8.01)	0.421	1.92(0.44-8.43)	0.387
20-39	0.7(0.32-1.54)	0.369	1.03(0.37-2.82)	0.959	1.04(0.38-2.84)	0.937
>=40	1.19(0.51-2.78)	0.686	1.41(0.49-4.06)	0.528	1.5(0.52-4.31)	0.447
Pack years						
pack years category	1(0.99-1.01)	0.787	1.01(0.99-1.02)	0.302	1.01(0.99-1.02)	0.256
0						
<15	1.53(0.65-3.57)	0.327	1.65(0.58-4.69)	0.347	1.7(0.60-4.83)	0.321
15-30	0.21(0.03-1.56)	0.128	0.31(0.04-2.48)	0.27	0.32(0.04-2.50)	0.275
>=30	1.13(0.56-2.30)	0.736	1.55(0.59-4.03)	0.372	1.64(0.63-4.24)	0.309
Age started smoking group						
non-smoker	reference					
<18	1.42(0.57-3.57)	0.453	1.91(0.62-5.86)	0.257	2(0.66-6.09)	0.221
18-25	0.9(0.41-1.99)	0.792	1.39(0.50-3.90)	0.529	1.43(0.51-4.00)	0.492
>=25	0.76(0.28-2.11)	0.602	0.78(0.22-2.81)	0.706	0.8(0.22-2.88)	0.738

Model 1, unadjusted model; Model 2, adjusted for age, sex, BMI, education, marital status, drinking, hypertension and CVD; Model 3, adjusted for age, sex, BMI, education, marital status, drinking, hypertension, CVD and COPD. Abbreviation: HR, hazard ratio; CI, confidence intervals.

Figure S1

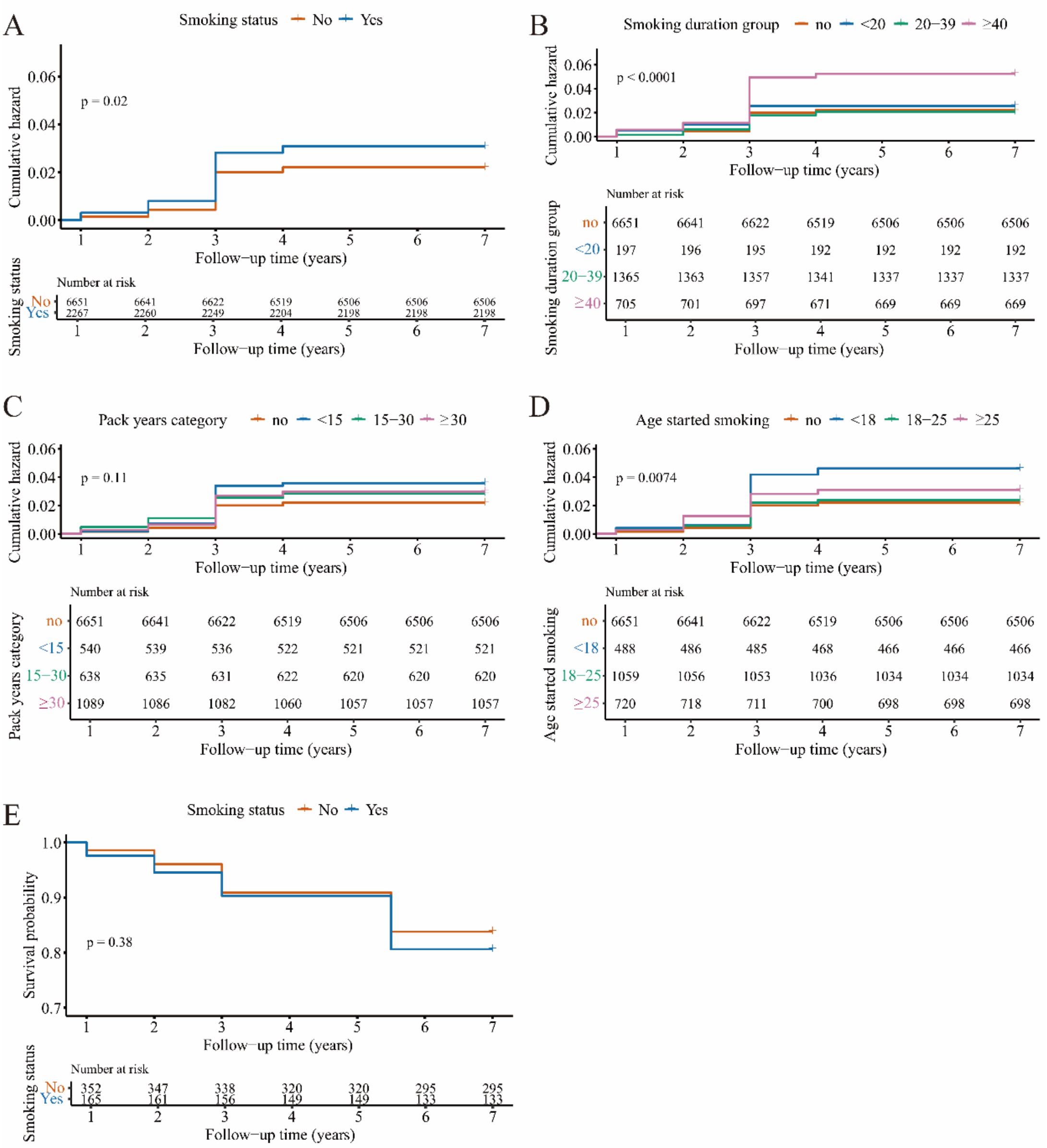

Figure S1. Nelson-Aalen cumulative hazard curves and Kaplan-Meier survival curve illustrating asthma incidence and all-cause mortality across different smoking behaviors in middle-aged and older adults. A-D, Nelson-Aalen curves for asthma incidence stratified by (A) smoking status, (B) smoking duration group, (C) pack-years category, and (D) age started smoking. E, Kaplan-Meier curve for all-cause mortality among asthma patients according to smoking status. Overall group differences were assessed using the log-rank test.

© 2025 Fu T. et al.
